# Gaze-angle dependency of pupil-size measurements in head-mounted eye tracking

**DOI:** 10.3758/s13428-021-01657-8

**Published:** 2021-08-04

**Authors:** Bernhard Petersch, Kai Dierkes

**Affiliations:** Pupil Labs, Sanderstraße 28, 12047 Berlin, Germany

**Keywords:** Pupillometry, Pupil foreshortening error, PFE, Eye tracking, 3D eye model, Corneal refraction

## Abstract

Pupillometry - the study of temporal changes in pupil diameter as a function of external light stimuli or cognitive processing - requires the accurate and gaze-angle independent measurement of pupil dilation. Expected response amplitudes often are only a few percent relative to a pre-stimulus baseline, thus demanding for sub-millimeter accuracy. Video-based approaches to pupil-size measurement aim at inferring pupil dilation from eye images alone. Eyeball rotation in relation to the recording camera as well as optical effects due to refraction at corneal interfaces can, however, induce so-called pupil foreshortening errors (PFE), i.e. systematic gaze-angle dependent changes of apparent pupil size that are on a par with typical response amplitudes. While PFE and options for its correction have been discussed for remote eye trackers, for head-mounted eye trackers such an assessment is still lacking. In this work, we therefore gauge the extent of PFE in three measurement techniques, all based on eye images recorded with a single near-eye camera. We present both real world experimental data as well as results obtained on synthetically generated eye images. We discuss PFE effects at three different levels of data aggregation: the sample, subject, and population level. In particular, we show that a recently proposed refraction-aware approach employing a mathematical 3D eye model is successful in providing pupil-size measurements which are gaze-angle independent at the population level.

## Introduction

Pupil size and its variation over time have long been recognized as powerful non-invasive metrics correlating with human cognitive processing (Mathôt, 2018). Today, pupillometry is an established research and diagnostic tool with applications in psychology (Laeng et al., 2012), neurology (Laeng & Alnaes, 2019), and medicine (Phillips et al., 2019).

Handheld pupillometers are readily available and routinely used in a clinical setting, e.g. for monitoring the pupillary light-reflex of patients. Other use cases, in particular in psychology and the behavioral sciences, are often better served by employing video-based eye-tracking systems, which next to gaze direction, also provide estimates of pupil size (Hutton, 2019). While remote eye trackers record the eyes of the subject using a stationary camera at a distance (e.g. mounted to a computer screen), head-mounted eye trackers feature near-eye cameras recording the eyes from close-up. Head-mounted eye trackers, in particular, hold the promise of giving access to pupil-size signals for subjects which are moving freely in real-world environments.

Video-based approaches to pupillometry estimate pupil size based on a single or a series of eye images. The 3D pupil, i.e. the ocular opening in the center of the iris, in humans is approximately circular (deviations from circularity are discussed e.g. in Wyatt 1995). When a subject is recorded by a camera from an oblique angle, the resulting 2D pupil image, however, is close in shape to an ellipse^1^. At least three optical effects influence apparent pupil shape in a camera image:

- (i) *Perspective foreshortening* - Moving the 3D pupil away from the camera makes it appear smaller.
- (ii) *Foreshortening with gaze angle* - Tilting the 3D pupil relative to the camera makes it appear more elliptic.
- (iii) *Corneal refraction* - Bending of light rays, occurring naturally at corneal interfaces, magnifies and distorts the image of the 3D pupil in a nonlinear fashion.

In the context of pupillometry, the combined effect of (i)-(iii) is often referred to as *pupil foreshortening error* (PFE), where “error” refers to the fact that any of these three factors, when not appropriately accounted for, can lead to incorrect pupil-size estimates.

**Fig. 1:**
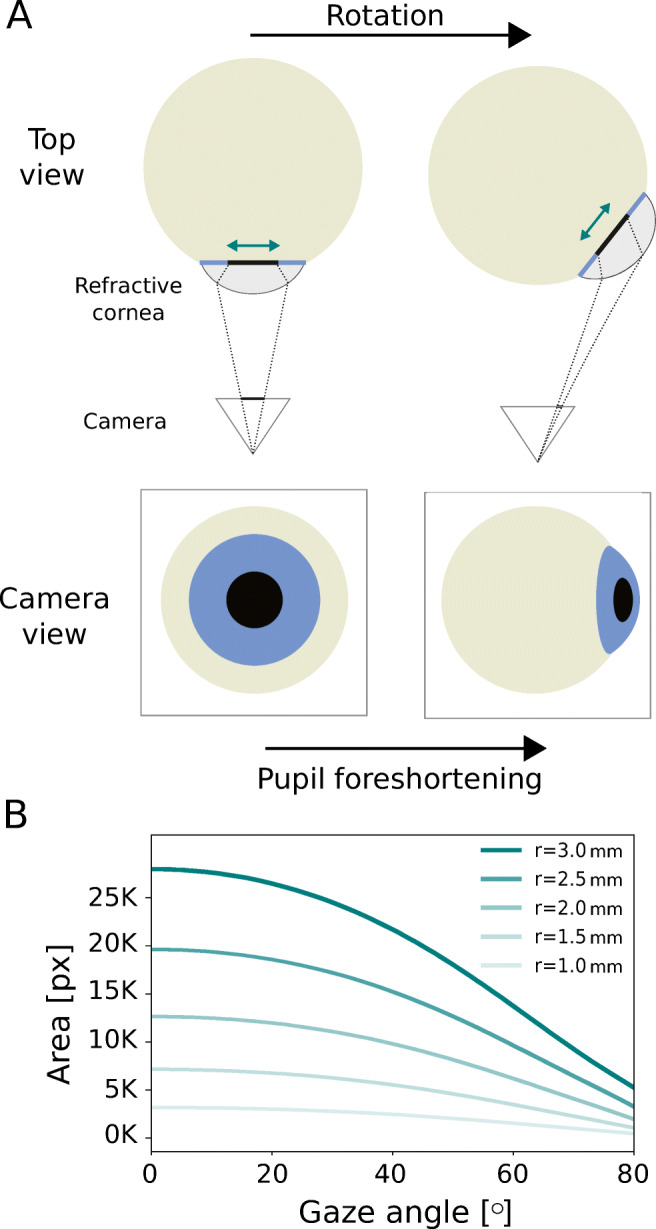
**Pupil foreshortening error (PFE).** **A** When imaging the 3D pupil by means of a near-eye camera (top view), the apparent shape of the 2D pupil (camera view) depends on the rotational state of the eyeball and the refractive properties of the cornea and aqueous humour. Under rotations of the eye, a 3D pupil of constant diameter (green arrows in top view) maps to 2D pupil images with non-constant geometric properties such as area. **B** Area of the 2D pupil in [px] as a function of gaze angle for various choices of the 3D pupil radius *r* in [mm]. The data shown was obtained by analyzing synthetic eye images such as shown in A, in particular modeling corneal refraction (see Methods)

To illustrate the detrimental effect of PFE on pupillometry results, consider pupil size is estimated by measuring the area of the apparent 2D pupil in image space. Furthermore, assume an eye with constant pupil size, which is recorded by a stationary camera in close proximity to the eye (see Fig. 1A). Due to foreshortening with gaze angle, a rotation of the eye away from the camera results in a decrease of apparent pupil area and thus of estimated pupil size (see Fig. 1B). In the extreme case of recording the eye from a sufficiently oblique view, apparent pupil area altogether reduces to zero. Thus, due to PFE, pupil-size estimates based on apparent pupil area exhibit a pronounced dependency on gaze angle. Note, the exact dependency is also shaped by (i) and (iii).

One option to minimize the impact of PFE is to design experiments which only employ relatively small static gaze targets appearing always at the same position within the test subject’s field-of-view. In such a scenario, relative pupil-size measurements can still be used to assess pupillary dynamics. Many pupillometry experiments, however, do necessitate dynamically changing gaze directions, as e.g. studies of visual search and reading tasks. Indeed, in these cases pupil-size estimates based on apparent pupil area can introduce errors comparable in magnitude to the cognitively induced pupil-size changes to be measured (Gagl et al., 2011). It is thus desirable to develop approaches to the measurement of pupil size, which do not exhibit any systematic variation with gaze angle.

A perfect measurement method would generate pupil-size measurements bearing no correlation with gaze angle for any given eye image, i.e. on the *sample level*. Given the variability in eye physiology and the number of simplifying assumptions necessary for deriving pupil size from camera images, at present such a tool appears out of reach. Pupillometry experiments, however, often involve extensive averaging over repeated trials of the same experiment (Sirois and Brisson, 2014; Laeng & Alnaes, 2019), either with the same subject or a population of subjects. Devising measurement methods which are independent of gaze angle on average, either on the *subject level* and/or the *population level*, thus is a relevant and pressing pursuit.

Several pioneering works have raised awareness for PFE-based confounds in pupillometry research using *remote* eye trackers and have proposed strategies for correcting for gaze-angle induced artifacts (Gagl et al., 2011; Brisson et al., 2013; Hayes & Petrov, 2016). Even though Pomplun and Sunkara (2003) have shared first insights on PFE in the *head-mounted* case, an in-depth analysis for this scenario is still lacking. We close this gap and shed light on the effect of PFE on pupil-size measurements by means of head-mounted eye trackers, more specifically, of the widely used Pupil Core headset sold by Pupil Labs (Kassner et al., 2014). Our contributions include the following:

- We present results of an experimental study, designed to gauge the extent of gaze-angle dependency in three approaches to pupil-size measurement.
- We provide experimental data, indicating gaze-angle dependency is critically shaped by subject-specific factors.
- In particular, we show that the refraction-aware approach proposed by Dierkes et al., (2019) successfully eliminates gaze-angle dependencies at the population level.
- In the framework of a simulation study, we show that the results of our experimental study are in quantitative agreement with theoretical predictions.

## Related work

The present study is related to (i) experimental and theoretical work with regard to the effects of ocular optics on apparent pupil shape as recorded by near-eye cameras and (ii) techniques proposed for assessing and correcting gaze-angle dependent errors of pupil-size measurements in remote and head-mounted eye trackers.

### Apparent pupil shape

Early experimental quantification of apparent pupil shape as a function of viewing angle has been contributed by Spring and Stiles (1948) and Jay (1962). For a more recent example, see the work by Mathur et al., (2013). Making use of photographic techniques, these studies map out the systematic changes in geometric properties of the apparent pupil when viewing a 3D pupil of constant radius at varying angles. Their data shows that apparent pupil shape can not be accounted for by assuming a mere perspective projection of the 3D pupil circle into the image plane. In particular, the rate of pupil foreshortening was consistently found to be lower than implied by this simplifying assumption. Instead, apparent pupil shape critically depends on effects introduced by refraction of light rays at corneal interfaces. From a theoretical perspective, the repercussions of corneal refraction on apparent pupil shape have been investigated by Fedtke et al., (2010) and Aguirre (2019). Employing a raytracing approach in the framework of the Navarro eye model (Navarro et al., 1985), these studies demonstrate that the experimental data on apparent pupil shape can be largely accounted for when refraction at corneal interfaces is considered. While Fedtke et al. furthermore provide an in-depth analysis of the 3D shape of the so-called entrance pupil, Aguirre presents arguments for resolving remaining discrepancies between theory and experimental observation by means of introducing non-circular 3D pupils into his theory. For an experimental study analyzing the non-circularity of the 3D pupil see the work by Wyatt (1995).

All works mentioned provide fundamental insights as to how for a given 3D pupil, ocular optics determines apparent pupil shape as recorded by a near-eye camera. They do not, however, furnish any means for solving the inverse problem, i.e. for inferring pupil size from given eye images. It is the latter question that we are addressing in the present study.

### Gaze-angle dependency of pupil-size measurements

In a review, Sirois and Brisson (2014) claim that gaze-angle induced pupil-size “*errors are systematic, and relatively easy to assess and correct*”. While errors are certainly systematic, they depend in a complex way on the pupil radius itself, the particular imaging geometry, and individual physiological parameters of the test subject’s eyes. This makes their assessment in a real-world setting a challenging and laborious task. This becomes apparent from the work of several groups (Gagl et al., 2011; Brisson et al., 2013; Hayes & Petrov, 2016), which all have proposed ways for mitigating gaze-angle induced pupil-size measurement errors in commercially available remote eye-tracking systems.

Gagl et al., (2011) studied the dependence of the pupil-size output of the SR-Research Eyelink 1000 remote eye tracker on horizontal gaze angle. To this end, the authors recorded pupil-size data from (i) test persons performing an *effortless* z-string reading task and (ii) an artificial eye with constant pupil radius that was rotated horizontally to mimic the horizontal eye movements occurring in (i). In both sets of experiments, pupil size was assumed constant over time. By measuring consistent systematic deviations in both cases, the authors showed that pupil-size estimates obtained with the Eyelink 1000 are indeed prone to gaze-angle induced artifacts. As a remedy, Gagl et al. proposed correction functions based on polynomial least-square fits to the data obtained in (i) and (ii). They further argued for the efficacy of their approach by successfully correcting a third set of pupil-size data, this time recorded during an *effortful* sentence reading task. More specifically, they could show that only after applying their correction functions, their data was consistent with findings reported in the literature for similar cognitive tasks.

Brisson et al., (2013) investigated the effect of *both* horizontal and vertical gaze direction on pupil-size estimation using *three* remote eye-tracking systems (Tobii T120/X120, Eyelink 1000). They recorded data from test subjects performing an effortless task, consisting of the pursuit of a dot describing elliptical movement on a display screen. Given the even illumination and the negligible cognitive load induced, they assumed an approximately constant pupil size. Systematic deviations correlating with screen position were found in the measurements by all three systems. Brisson et al. further showed that 10-20 % of the observed pupil-size variation could be explained by a linear regression of pupil size from horizontal and vertical screen coordinates, with “*the remaining variation* [being] *intra- and inter-individual variation*”.

Hayes and Petrov (2016) took a similar route as Gagl et al., in that they mapped gaze-angle dependency of pupil-radius estimates utilizing artificial eyes without cornea. Working with the EyeLink 1000, they extended earlier results by employing *three* artificial eyes, each with a different fixed pupil size. In addition, they systematically measured deviations in estimated pupil size as a function of gaze position across three experimental layouts, varying the relative distances between the eye-tracking camera, eye, and display. Making simplifying assumptions specific to the remote case, e.g. that eyeball diameter is negligible relative to the distance between eye and recording camera, they derived a geometric model of PFE for eyes without cornea. They showed that their model is able to reduce the root mean squared error of pupil size measurements of the artificial eyes by 82.5 % when the model parameters were pre-set to the physical layout dimensions, and by 97.5 % when numerically optimizing the model parameters to fit the measured pupil size errors. Finally, they suggested to incorporate empirical foreshortening functions as measured on dilated human eyes by Mathur et al., (2013) into their model to account for refraction effects in real eyes.

Each of the seminal works outlined above - which have also been discussed by Mathôt et al., (2018) - has devised ways for generating phenomenological correction functions to at least partially eliminate gaze-angle induced errors in pupil-size measurements. Each has certain merits and detriments. While the use of artificial eyes offers the possibility of generating measurements for known ground-truth pupil sizes, artificial eyes also tend to be anatomically crude. In particular, in case they lack an optically realistic cornea they will not reflect contributions of corneal refraction to PFE. Artificial eyes comprising elements mimicking the human cornea to some extent are commercially available, but usually designed for gaze estimation instead of pupillometry quality assurance. Even the more complex ones lack adjustable pupil size and refractive properties of the materials used for construction do not necessarily closely match physiological values (Wang et al., 2017). Mapping gaze-angle dependencies with human subjects in scenarios that approximate constant pupil size over time, potentially circumvents this problem. However, at the cost of an unknown ground-truth pupil size, limiting potential correction schemes to being relative multipliers only. In both cases, correction functions are specific to a certain experimental setup, necessitating new measurements whenever the setup is changed. Most relevant for the current study, it is questionable whether the assumptions and approximations made for the remote case port to the head-mounted scenario.

The head-mounted case was first studied by Pomplun and Sunkara (2003). Employing an EyeLink-II system, they were able to show that the area-based pupil-size estimate provided by the eye tracker was indeed subject to a systematic gaze-dependent variation (cf. our simulation data shown in Fig. 1B). The authors proposed a calibration routine for correcting for the observed dependency in a person-specific manner. They argued for the efficacy of their approach in a second set of experiments, in which an increase in cognitive load during a series of screen-based tasks could be correctly detected on the basis of gaze-angle adjusted pupil dilations.

In more recent work, Dierkes et al. (2018, 2019) study the effect of gaze angle on pupil-size estimates employing a Pupil Core eye tracker as available from Pupil Labs. Taking a different approach, the authors made use of synthetic eye images in order to map the gaze-angle dependency of pupil-radius estimates as provided by Pupil Capture, the open-source software used to operate the headset. Pupil Capture employs a model-based approach to gaze and pupil-size estimation which is closely based on the work by Świrski and Dodgson (2013). By means of a raytracing pipeline, Dierkes et al. generated eye images within the framework of the LeGrand model (Le Grand, 1957), a widely used description of the average human eye. In particular, this allowed for accounting for corneal refraction in a realistic way. Providing such a framework replaces the burden of manual measurements of human and/or artificial eyes with comparably cheap and fast computer simulations, which can be easily re-run for different hardware and imaging/camera set-ups as well as eye physiologies. Subjecting the simulated images to the pupil-size estimation algorithm, the authors established a mapping between ground-truth pupil size (which is always known in simulations) and measured values. In particular, they found that pupil-size estimates deviated by almost 10% when the eye was rotated away from the camera by about 60^∘^. Based on the data generated, polynomial correction functions were fit, which subsequently were used to successfully correct for gaze-angle dependencies in the synthetic pupil-size data. Preliminary real-world data was presented in Dierkes et al., (2018), qualitatively confirming that the ratio of uncorrected to corrected pupil size followed the trend expected from the synthetic data.

Here, we expand on the work by Dierkes et al. in several ways. By means of controlled experiments, which were designed to realize an approximately constant pupil size over time, we provide a direct quantitative verification of their approach in correcting for gaze-angle dependencies at the population level. Unlike the method described by Pomplum and Sunkara, no calibration is needed to achieve this result. We further gauge the method proposed by Dierkes et al. against two other techniques, consisting of (i) estimating pupil size by the major axis of the apparent pupil ellipse and (ii) the pupil-size estimate as provided by the uncorrected Świrski model. In particular, we shed light on the ability of all three methods to provide pupil-size estimates which are independent of gaze angle on the sample, subject, and population level. By means of synthetically generated eye images, we furthermore show that our experimental findings are in quantitative agreement with predictions based on a widely used model of ocular optics.

## Methods

In this section, we describe the methods, experimental protocols, and data analysis techniques used in this study. We start by recapitulating the three evaluated methods for measuring pupil size. After providing details with regard to the protocol for recording real-world data in two different constant pupil-size scenarios, we briefly introduce the ray-tracing pipeline employed for generating synthetic eye images. In particular, we outline the analysis and data aggregation steps performed for extracting pupil-size data both from real-world recordings and synthetic eye images on the sample, subject, and population level.

### Pupil-size measurement methods

Video-based pupillometry methods can be classified (i) as to whether they explicitly account for gaze angle and/or corneal refraction, (ii) as to whether they output a physical pupil aperture stop size in [mm] or merely provide a value in arbitrary units, and (iii) as to how many physiological parameters are necessary for their full specification.

#### Major axis: 2D-0p

**Fig. 2:**
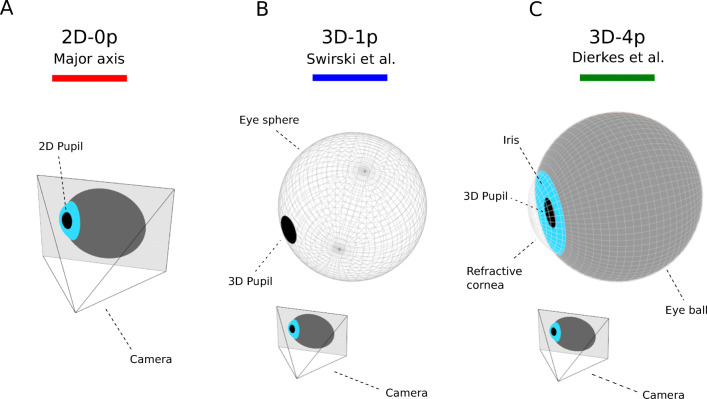
**Approaches to measuring pupil size in head-mounted eye trackers.** **A** The major axis of an ellipse fit to the 2D pupil as appearing in an eye image provides a pupil-size estimate in pixels. Since this approach does not presume any physiological parameters, we refer to it as 2D-0p. **B** The model-based approach by Świrski et al. stipulates the 3D pupil to be a circle tangent to a rotating eye sphere. It estimates pupil size as the radius of the 3D pupil circle and is reported in units of length (typically in [mm]). As it depends on the choice of a single physiological parameter (radius of the eye sphere), we refer to it as 3D-1p. **C** The model-based approach by Dierkes et al. utilizes the LeGrand eye model. In particular, it captures refraction effects occurring at corneal interfaces. Pupil size is estimated as the radius of the 3D pupil and is reported in units of length (typically in [mm]). As it relies on four physiological parameters (eye ball radius, iris radius, corneal radius, and refractive index of the cornea), we also refer to it as 3D-4p. Colored bars in each panel denote the color used in subsequent plots of results obtained with the respective method

The first method we consider derives a pupil-size measure directly from the shape of the 2D pupil in a given eye image (see Fig. 2A). The shape of the 2D pupil is commonly approximated by an ellipse. As shown in the Introduction, the area of the pupil ellipse is strongly affected by PFE. A geometric measure expected to depend less strongly on gaze angle is the length of its major axis. The term *major axis* shall therefore in the following signify the length of the major axis of the pupil ellipse in [pixels], suitably fitted to the region corresponding to the 2D pupil within a given eye image (see examples in Fig. 4).

This image-immanent 2D method for pupil-size measurement requires no physiological input parameters. We therefore also refer to it as *2D-0p*. Note, no explicit strategies for accounting for corneal refraction nor for changes in gaze angle are employed by this method.

#### 3D Eye model without cornea: 3D-1p

Reporting the length of the major axis of the pupil ellipse results in a pupil-size output in units of [pixels]. As pointed out by several authors, pupillometry experiments should preferably report pupil size in [mm] (see e.g. Beatty and Lucero-Wagoner (2000), Kelbsch et al., (2019), and Köles (2017)). By fitting a mathematical 3D eye model to video observations of the eye, so-called model-based approaches allow for deriving pupil-size measures in actual physical units. As a second method for measuring pupil size, we will therefore consider the model-based approach proposed by Świrski and Dodgson (2013).

Świrski et al. model the eye as comprising an eye sphere of fixed radius, with the 3D pupil being a circle of variable size tangent to it (see Fig. 2B). While changes in gaze angle correspond to rotations of the eye sphere around its center, changes in pupil dilation correspond to changes in the radius of the tangent pupil circle. Given the state of the eye model as well as the pose and intrinsics of a camera, the Świrski model predicts the shape of the pupil ellipse as appearing in an image taken by the camera. To this end, it assumes the 3D pupil circle is mapped to the image via a perspective projection with a pinhole camera. In particular, lacking a cornea, the Świrski model does not account for corneal refraction.

Given a series of pupil ellipse observations under varying gaze angles, the corresponding 3D location of the eye sphere in the coordinate system defined by the recording camera is estimated by solving a nonlinear optimization problem. After the eye sphere is located, the current pupil radius is estimated based on the pupil ellipse in a given eye image as follows. Essentially reversing the imaging operation, in a first step, the pupil ellipse is “unprojected” to find the 3D circle of radius *r* = 1 mm which under projection to the camera would yield the observed pupil ellipse. In this step a prior measurement of the intrinsics matrix and distortion coefficients of the camera can be employed to correct for center shift (principal point offset) and image distortion, if any. In a second step, the resulting 3D circle is then scaled along the unprojection cone until it lies tangent to the eye sphere estimated before. The scaling factor is the output pupil radius in units of [mm]. Note, the optical axis of the eye is also determined, since it corresponds to the line connecting the center of the scaled 3D circle and the center of the eye sphere.

The size of the eye sphere is the sole physiological parameter which is used in the Świrski model. We therefore also refer to it as *3D-1p*.

#### 3D Eye model with cornea: 3D-4p

While the method by Świrski et al. inherently takes varying gaze angles into account, it does not actively model corneal refraction. Since the image of the pupil is distorted by the refraction effect of the cornea, unprojection of the resulting pupil ellipse does not yield the correct 3D pupil circle. As already indicated in “Gaze-angle dependency of pupil-size measurements”, Dierkes et al., (2019) have presented a model-based technique for determining 3D eyeball position, optical axis, and pupil size from video images, which explicitly corrects for corneal refraction. Their approach constitutes the third method considered in this study.

The main idea underlying the method by Dierkes at al. is to derive correction functions to be applied to predictions by the Świrski model. To achieve this goal, they employ synthetically generated eye images based on the LeGrand eye model (Le Grand, 1957), a widely used approximation of the physiology and optics of the human eye (see Fig. 2C). The LeGrand eye model is characterized by four physiological parameters: eyeball radius, iris radius, cornea radius, and the refractive index of the cornea. Assuming a realistic imaging setup, a large number of eye images is raytraced, varying eyeball position with respect to the camera, as well as gaze angle and pupil radius, all within physiologically plausible ranges. Subjecting the resulting images to the algorithm proposed by Świrski et al., they obtain corresponding tuples of measured and ground truth eyeball positions, gaze vectors, and pupil sizes. Multivariate polynomial regression is then employed to fit a correction function, which can be applied in real-time to similar tuples measured by means of the Świrski model on real world data.

As this method employs as many physiological parameters as the underlying LeGrand eye model, i.e. four, we refer to this approach also as *3D-4p*. Estimated pupil size is reported in units of physical length, more specifically [mm]. Note, this approach in particular accounts both for the effects of corneal refraction as well as changes in gaze angle.

Table 1 summarizes key properties of the three pupil-size measurement methods employed. Unless stated otherwise, in the following whenever we refer to *pupil radius* and *corrected pupil radius*, this signifies a pupil radius in [mm] *as measured* by the methods presented in “3D Eye model without cornea: 3D-1p” and “3D Eye model with cornea: 3D-4p”, respectively. Note that this is in contrast to the *ground-truth* pupil size, which for an *in vivo* scenario on independent grounds is per se unknown.

**Table 1:** Properties of the three pupil-size measurement methods employed in this study

Method	Physiological	Output	Gaze angle/
	parameters	units	Refraction
2D-0p	-	[pixels]	-/-
3D-1p	Eye-sphere size	[mm]	+/-
Świrski and Dodgson (2013)			
3D-4p	Eyeball radius	[mm]	+/+
Dierkes et al.,	Iris radius		
2019	Cornea radius		
	Refractive index		

### Experiments - Real world data

Ground-truth pupil size is unavailable when performing *in vivo* measurements. This limitation prevents obtaining a direct verification of pupillometry accuracy for any method under evaluation. Gaze-angle dependency, however, provides an indirect metric for assessing the success of a given method in providing meaningful pupil-size measurements. The question we seek to answer quantitatively is therefore: how do pupil-size measurements obtained via the three methods outlined above perform in terms of this metric? To this end, we propose two protocols for *in vivo* measurements, both designed to reduce pupil-size fluctuations to a physiological minimum. In this way, we approximate two constant pupil-size scenarios, once producing a fairly constricted pupil, once a maximally dilated one. Given a perfect measurement method, estimated pupil size in these scenarios would be constant over time also upon changes in gaze directions, or at least only show variations which i) do not exceed those of reference periods with a static gaze and ii) do not correlate with gaze-angle variation.

#### Bright environment

**Fig. 3:**
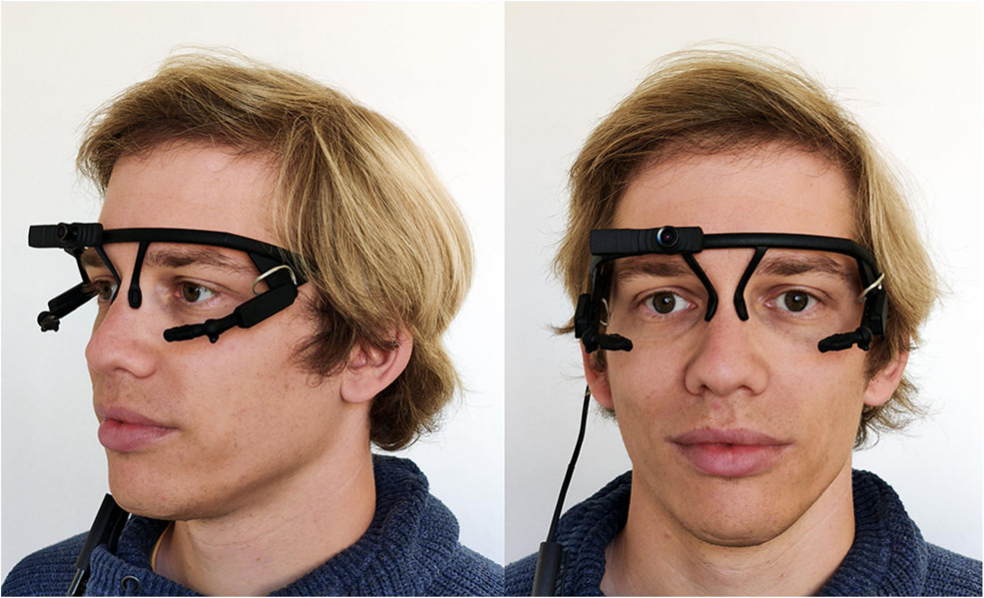
**Pupil Core binocular headset.** The head-mounted eye tracker sold by Pupil Labs features two near-eye cameras, one per eye. A forward-facing scene camera can be used to record the scene in front of the subject (not employed in this study). The Pupil Core headset was connected via USB to a laptop running the Pupil Capture software

In a first series of experiments, eye videos of the left and right eye of *N* = 16 test subjects were recorded at 120 fps in 400x400 pixel resolution using a standard binocular Pupil Core headset (see Fig. 3), using the open-source software Pupil Capture (version 1.21.202). Of the 16 test subjects, 13 were myopic (three of them only slightly, with spherical correction <= 0.5 dioptre), the remaining three were emmetropic. Five to six recordings per subject were made, each lasting 75 s. Recordings were performed in a windowless room. Subjects were standing at a distance of about 30 cm from an evenly painted, non-glossy white wall, facing the wall which carried a small (approximately 1 cm^2^) colored sticker as a fixation aid. The sole sources of light were two 500 W lamps on tripods, each aimed at the wall from a position approximately 1 m laterally and 1 m dorsally of the subject, thus producing a fairly even, brightly lit wall.

Prior to recording data for a given test subject, eye cameras were adjusted and the subject was instructed not to touch the headset during the recording, in order to minimize the occurrence of headset slippage.

Subjects were instructed to keep focusing on the colored sticker at all times. Each recording comprised an initial phase of 15 s of static straight ahead gaze, which provided time for the eyes to accommodate to the brightness level of the wall. This period was followed by 60 s of slow, random head rotations (referred to as *sweep period*). Since subjects maintained fixation on the sticker, this ensured that eye positions and rotational states with respect to the wall and thus to the diffuse but bright environment remained approximately constant, while sampling a diverse range of physiologically accessible gaze angles with respect to the headset-mounted eye cameras. In this way, we sought to create a situation in which pupil-size changes were reduced to a physiological minimum, i.e. being approximately constant with a fairly *small* pupil size. Recordings with each subject were performed in close succession, with no more than 60 s time in between recordings. Recordings were deliberately held short in order to avoid fatigue, a drift in pupil size, and to minimize headset slippage.

#### Dark environment

In a second series of experiments, eye videos of the left and right eye of the same *N* = 16 test subjects were recorded at 120 fps in 400x400 pixel resolution using the same binocular Pupil Core headset and Pupil Capture version. Again, five to six recordings per subject were made, each lasting 120 s. Recordings were performed in a light-tight room, in which no light sources were discernible even after 300 s of adaptation. Subjects were thus submerged in complete darkness and no fixation on any particular distance or target was possible. As compared to the bright environment, the duration of the initial static gaze period was increased in order to take the eye’s slower accommodation to darkness into account. Subjects were instructed to keep looking straight ahead at all times. Each recording comprised 60 s of static straight ahead gaze, followed by 60 s of slow, random head rotations (sweep period), while maintaining an approximately straight ahead gaze, just as in the bright environment. This way, we sought to create a situation in which pupil-size changes were reduced to a physiological minimum, this time, however, with a fairly *large* pupil size.

Summing bright and dark environment experiments, 233 individual recordings were made. Since always both eyes were recorded, a total of *n* = 2 ⋅ 233 = 466 monocular eye recordings were obtained, resulting in approximately 4 ⋅ 10^6^ individual image frames, which formed the basis of our data analysis.

#### Multi-day recordings

To probe whether pupil-radius estimates for a given subject correlate with gaze angle in a manner consistent from recording session to recording session, for two subjects experiments on three consecutive days were performed. More specifically, on each day recordings were made for each subject in the bright environment.

### Experiments - Synthetic data

In order to study the gaze-angle dependency of pupil-size estimates from a theoretical vantage point, we employ the raytracing pipeline presented by Dierkes et al., (2019) (see also “Gaze-angle dependency of pupil-size measurements” and “3D Eye model with cornea: 3D-4p”). More specifically, we generated synthetic eye images within the framework of the LeGrand eye model (Le Grand, 1957) (see Fig. 2C). During the raytracing operation, for each pixel in the image, a simulated light ray emanating from the center of an idealized pinhole camera is cast towards the eye model. Pixel color is determined by means of distinguishing four cases: (i) the simulated light ray passes the eye without intersection (resulting in a white pixel), (ii) it hits the eyeball (resulting in a beige pixel), or it hits the cornea, in which case it is refracted according to Snell’s law and continues its path until it either (iii) hits the iris (resulting in a blue pixel) or (iv) enters the pupil (resulting in a black pixel). Note that within the scope of the LeGrand eye model, cornea and aqueous humour are assumed to form a continuous medium with a uniform refractive index *n*_ref_. In the generation of all synthetic eye images, we set *n*_ref_ = 1.3375 (Guestrin and Eizenman, 2006). Examples of generated eye images are shown in Figs. 1A and 2.

Two sets of simulations were performed, one with strictly constant pupil size and another one including pupil-size fluctuations of realistic amplitude. For the first set, in total, *n* = 800 simulated eye recordings were generated, mimicking our experiments in the bright and dark environment. For each simulation run, the position of the eyeball in camera coordinates was randomly chosen from a range of positions consistent with realistic setups of the Pupil Core headset. To reflect the physiological variability of human eyes, the eyeball radius, iris radius, and corneal radius of curvature were chosen randomly, each from a normal distribution with realistic mean and standard deviation (see Table 2). A constant pupil radius was randomly chosen for each simulation run from a uniform distribution between 0.5 mm and 4.5 mm, thus covering the full range of pupil radii typically encountered in humans (see e.g. Sirois and Brisson, 2014). Utilizing the above parameter values, eye images corresponding to 500 random gaze angles from a physiologically plausible range were raytraced. Images with only partially visible 2D pupil (rarely occurring for extreme eyeball positions in combination with extreme gaze angles) were discarded. The images generated in one simulation run thus correspond to an individual sweep period as recorded in our real world experiments.

**Table 2:** Mean and standard deviation of normal distributions assumed in the generation of synthetic eye images for capturing the variability of physiological parameters in the LeGrand eye model

Parameter	Mean	Std	Ref.
Eyeball radius	12.0 mm	1.0 mm	Bekerman et al., (2014)
Cornea radius	7.81 mm	0.24 mm	Montalbán et al., (2013)
Iris radius	5.57 mm	0.28 mm	Aguirre (2019)

For the second set of simulations, while keeping all other steps as described above, pupil size for each of the 500 eye images in a simulation run was modulated by a random factor independently drawn from a normal distribution with mean *μ* = 1 and a standard deviation drawn from a distribution according to the one shown in Fig. 7 (Major axis). In this way, relative pupil size over each simulated recording fluctuated with a standard deviation consistent with estimated amplitudes of pupil-size changes in our experiments (see “Sample level”). The same data processing and analysis as detailed for the real world data in the next section was applied to the synthetic data, unless stated otherwise.

### Data processing & Analysis

Pupil contours were extracted from all real-world images by means of the open-source 2D pupil detector implemented in Pupil Capture (Pupil Labs, 2020a). Resulting pupil detections comprise an ellipse fit to the 2D pupil and thus, in particular, provide the length of its major axis without the need of further analysis. As the eye cameras used in the experiments showed negligible image distortion, pupil detections were only corrected for principal point offset using measured camera intrinsics.

From synthetic eye images, the 2D pupil can be trivially extracted by virtue of the corresponding pixel label obtained during raytracing. An off-the-shelf algorithm was employed to obtain the best-fitting ellipse (scikit-image dev team, 2020).

In order to calculate pupil radii in the framework of either of the two model-based methods (3D-1p and 3D-4p), in a first step the 3D position of the eye sphere and eyeball, respectively, needs to be estimated based on a time series of pupil detections. More specifically, we used the formulation proposed by Dierkes et al., which casts the involved minimization problem as a least-squares intersection of lines (Dierkes et al., 2019). This algorithmic approach is implemented in the open-source pye3d-detector Python package developed by Pupil Labs (2020b). For each recording, we determined the corresponding eye sphere and eyeball position for the left and right eye post-recording, based on eye-camera frames from the sweep period. To reduce the effect of erroneous pupil detections, only the 10 % frames with highest pupil-detection confidence entered the optimization. The confidence measure, a value ranging from 0.0 (lowest confidence) to 1.0 (highest confidence), was provided by the employed 2D pupil detector.

For synthetic eye images, 2D pupil segmentation is always equally confident. Thus, for each simulation run, eye sphere and eyeball position was estimated using all images generated during the respective run.

Given the estimates of eye sphere and eyeball position, uncorrected and refraction-corrected pupil radii were calculated for each eye image for both kinds of data (real-world and synthetic) as outlined in “Major axis: 2D-0p”, “3D Eye model without cornea: 3D-1p”, and “3D Eye model with cornea: 3D-4p”. For the model-based approaches, we employed functionality from the pye3d-detector Python package developed by Pupil Labs (2020b). The resulting time series, three per eye for each recording as well as for each simulation run, constitute our sample-level results.

For each recording and each of the three approaches to pupil-size measurement, we determined a baseline by obtaining the median of the respective pupil-size measure over all data samples from the sweep period with a confidence of at least 0.6. Note, the experimental setup was designed to assure a pupil size that was approximately constant over time. The baseline value thus also serves as an estimate of the constant pupil size realized in each experiment. Real-world samples with a major axis deviating by more than 20 % from the corresponding baseline value were excluded from further analysis, since variations of such large amplitude could be considered erroneous pupil detections. This resulted in exclusion of less than 4 % of data samples. Analogous baselines were measured for all simulation runs. No measurements needed to be discarded, however, from the synthetic data. In order to make correlations in pupil size with gaze angle comparable between subjects and recording environments, each time series was normalized by division with the respective baseline value. We refer to the resulting values as relative pupil sizes.

**Fig. 4:**
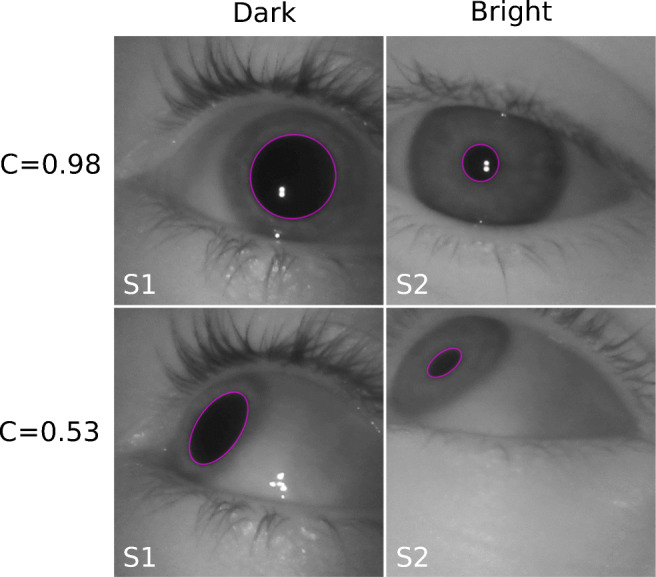
**Sample eye images.** Typical images as obtained in the dark (left column, subject 1) and bright environment (right column, subject 2). Fit pupil ellipses are shown as pink lines. Rows comprise example images with identical pupil ellipse circularity *C* as indicated. Note that changes in gaze angle (cf. upper vs. lower row) induce corresponding changes in pupil ellipse circularity

We ultimately seek to shed light on the variation of pupil size as a function of gaze angle. Both model-based methods, for each eye image also provide a gaze-angle estimate. The respective estimates, however, depend both on model assumptions as well as the choice of model-specific parameters. Striving for a more objective measure, we decided to use circularity *C* of the 2D pupil ellipse instead. More specifically, circularity is defined as the ratio of minor and major axis of the 2D pupil ellipse. Circularity values thus range from 0 to 1, with 1 corresponding to a perfectly circular pupil image (see Fig. 4). The latter case is observed when looking straight at the camera, with the eyeball being positioned on the camera’s optical axis (see Fig. 1A, left synthetic eye image). With increasing angle between the optical axis of the eye and the camera, respectively, circularity of the 2D pupil ellipse decreases (see Fig. 1A, right synthetic eye image). For off-axis eyeball positions, i.e. when rotational symmetry is broken, circularity can differ slightly for identical gaze angles. Since on theoretical grounds these deviations are expected to be small, however, we decided to use circularity of the 2D pupil ellipse as a convenient proxy for gaze angle. In particular, this strategy allowed us to average pupil-size data from different but equivalent gaze directions.

More specifically, for each subject, each eye, and each approach to pupil-size measurement, we performed the following data aggregation. Relative pupil sizes as obtained during the sweep period were binned according to circularity into ten bins of equal width, spanning the theoretical range from 0 and 1. The weighted mean over all recordings, both from the bright as well as the dark environment, was then calculated in each bin. As weight for each sample we used the corresponding pupil-detection confidence.

In the case of the synthetic data, each pupil-size sample contributed equally. To further make results comparable between subjects as well as simulation runs, the resulting values were normalized by dividing by the value of the bin corresponding to the highest circularity. On the subject level, per eye all recorded data was thus aggregated into normalized average relative pupil size as a function of circularity.

At the population level, in each bin we further calculated the mean and standard deviation of all curves obtained on the subject level.

For ease of reference, below we summarize the data processing steps in condensed form:

1. **Sample level**
  i) Detect pupil ellipses in all recorded eye images.
  ii) Estimate 3D eye sphere and eyeball position based on high-confidence pupil detections from sweep period of each recording.
  iii) Determine pupil size for each eye image.
  iv) Calculate baseline, i.e. median of pupil-size estimates during sweep period with pupil-detection confidence larger than 0.6.

2. **Subject level**
  i) Filter out all data samples with a pupil ellipse with major axis deviating from the corresponding baseline by more than 20 %.
  ii) For each eye, calculate the confidence-weighted mean of pupil sizes divided by baseline in ten circularity bins between 0 and 1. The mean is taken over all recordings for a given subject.
  iii) Normalize by average pupil-size value of the bin corresponding to the highest circularity, i.e. C between 0.9 and 1.0.

3. **Population level**
  i) Calculate mean and standard deviation over test subjects and left and right eyes within each bin.

## Results

### Sample level

Sample eye images recorded for two subjects in the dark and bright environment, respectively, are displayed in Fig. 4. Pupil ellipses as detected by Pupil Capture are shown (pink lines) with their circularity being indicated. As can be seen from the images, rotation of the eye with respect to the camera induces changes in the circularity of the observed pupil ellipse. In this study, we use circularity as a proxy for gaze direction, as motivated in the previous section. In particular, note the larger pupil dilation in the dark compared to the bright environment.

**Fig. 5:**
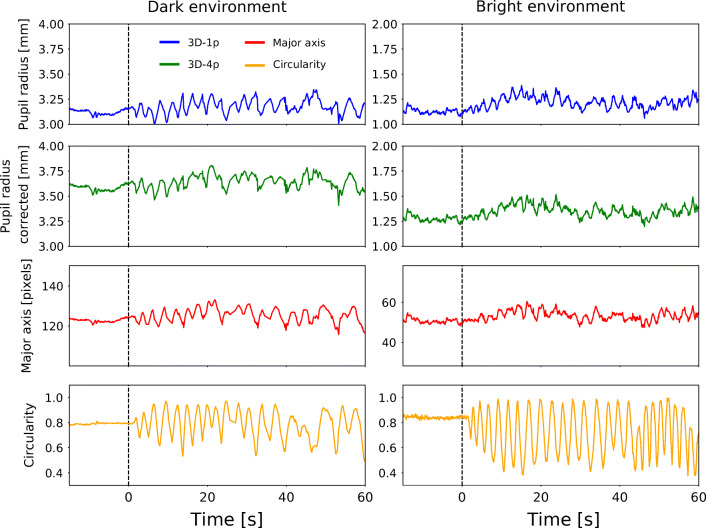
**Sample-level real-world data.** Time series of pupil-radius estimates as extracted from one of the dark (left column) and one of the bright environment recordings (right column). Next to results from the three measurement methods (top three rows, legend in top left panel), circularity of the 2D pupil is shown (bottom row). All data as a function of time in seconds, with *t* < 0 being the static gaze period and *t* > 0 comprising eye rotations (sweep period). Smaller absolute pupil size for the bright environment is apparent for all three methods (note the different scales used for the left and right column). Refraction correction *in* creases measured pupil size. This is mainly due to the fact that the uncorrected 3D-1p approach yields eye sphere positions which systematically lie too close to the camera (cf. (Dierkes et al., 2018)). During eye rotations in the sweep period (*t* > 0), all three pupil-size measures correlate to some extent with gaze angle as represented by circularity

Time series of pupil-size measurements obtained via all three methods are shown in Fig. 5 (blue, green, and red lines). More specifically, data from a typical monocular recording obtained in the dark and bright environment is presented in the left and right column, respectively. Note the different extent of the vertical axes used to present the data from the dark versus the bright environment. Also shown are corresponding circularity values (orange lines). Vertical dashed lines at time *t* = 0 s represent the transition from static straight ahead gaze to the sweep period.

In the sweep period (*t* > 0 s), oscillatory variations in circularity are apparent in both recordings. These variations provide evidence that the performed random head rotations were efficacious in inducing gaze-angle changes relative to the recording camera. A correlating variation can be discerned in the results of all three measurement methods, showing that to a certain extent all methods exhibit gaze-angle dependency on the sample level.

**Fig. 6:**
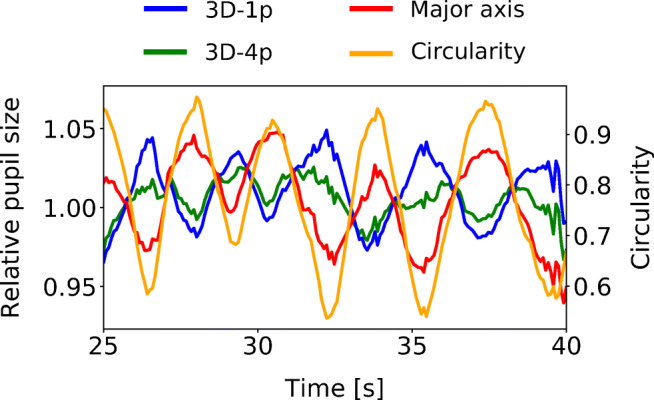
**Close-up of sample-level data.** Data corresponds to a section of the dark environment time series presented in Fig. 5. Shown are pupil-size measurements normalized by their respective baseline (uncorrected 3D-1p in blue, refraction corrected 3D-4p in green, and major axis in red) as well as circularity (orange). While correlation with circularity (and thus gaze angle) is not fully eliminated at the level of each individual pupil-size measurement, the refraction-corrected pupil radius provided by 3D-4p exhibits less deviations from unity than the other two methods, indicating a partial reduction in gaze-angle dependency

In order to further probe the observed correlation, in Fig. 6 we show in a single panel results from all three methods normalized to their respective baseline. More specifically, the data shown corresponds to the period 25 s < *t* < 40 s in the dark environment example shown in Fig. 5. Several observations can be made. While the major axis correlates positively with circularity, both model-based methods correlate negatively with it. Upon a rotation away from the camera, the major axis thus tends to underestimate pupil size relative to the value at *C* = 1, while both model-based approaches tend to overestimate it. As to their amplitude, major axis and 3D-1p exhibit variations of similar size. The amplitude of variation in the 3D-4p results in comparison appears reduced. While this reduction is indicative of the partial success of the employed refraction-correction scheme, it is not able to fully eliminate all gaze-angle dependency on the sample level.

**Fig. 7:**
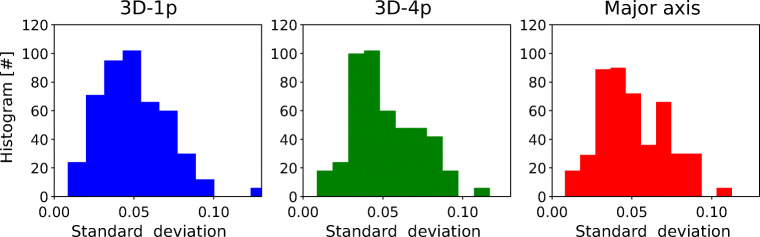
**Pre-sweep pupil-size fluctuations.** Shown are histograms of standard deviations of relative pupil size as calculated for each recording over the 10 s preceeding the sweep period. Recordings from both the dark and bright environment were used. Pupil-size fluctuations have a mean standard deviation of about 5%, irrespective of measurement approach

In addition to gaze-angle induced changes of measured pupil size, the example time series shown also exhibit pupil-size fluctuations on longer time scales. This becomes apparent in particular in the bright environment example, in which pupil size as measured by all three methods increases by about 10 % over the first 30 s shown. While not as obvious in the dark environment example, there is reason to suspect pupil-size fluctuations also in this case. Lacking a visible fixation target, in the dark environment the accommodative state of the eye is ill defined. In particular, we cannot exclude that the Pupil Near Response (PNR) - which entails pupil-size changes - was triggered at random points in time. In addition, it has been reported that in darkness and/or in the absence of direct external stimuli, the size of the pupil can fluctuate with frequencies around 0.5-1.0 Hz and amplitudes of the order of a few percent (Köles, 2017; Sirois and Brisson, 2014; Mathôt et al., 2018). In order to estimate the extent to which pupil size was fluctuating over the time course of our experiments, for all recordings and all three measurement methods, we calculated the standard deviation of relative pupil size over a time window of 10 s prior to the sweep period (see Fig. 7). Participants were instructed to hold a static gaze prior to the sweep period. These measurements were therefore not influenced by PFE. As the resulting histograms show, fluctuations relative to the baseline for all three approaches had standard deviations of up to 10%, with a mean of approximately 5%. Thus, while our experiments were carefully designed to provide controlled light conditions, due to physiological factors outside of our control, a constant pupil size in any given recording could only be approximately realized.

### Subject level

Physiologically induced pupil-size fluctuations bear no correlation with gaze direction. Aggregating pupil-size measurements in terms of pupil-ellipse circularity allows for integrating data from different but equivalent gaze directions as well as independent points in time. It thus provides a tool for averaging out pupil-size fluctuations that are uncorrelated with gaze direction. Note, we will further analyze the effect of fluctuating pupil size in “Population level” by means of our simulations of synthetic eye recordings.

**Fig. 8:**
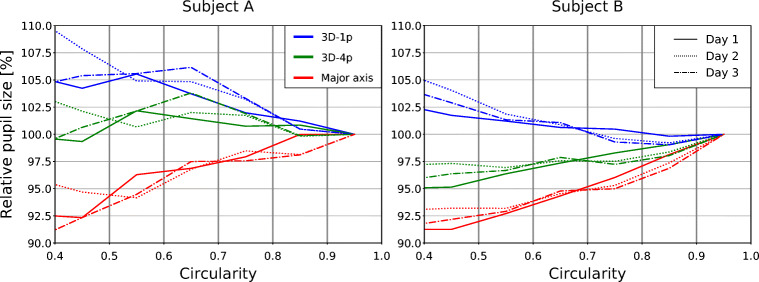
**Subject-level real-world data from different days.** Normalized average relative pupil size is shown as a function of pupil circularity for two test subjects (A, left; B, right) and all three measurement methods. For each subject, recordings in the bright environment were made on three different days (solid, dotted, and dashed lines). For both subjects, the major axis tends to underestimate pupil size with decreasing circularity (i.e. increasing gaze angle); 3D-1p tends to overestimate it. As for the refraction-corrected method 3D-4p, in the case of subject A it produces results which are notably less dependent on gaze angle as either of the two other methods. For subject B, 3D-4p tends to underestimate pupil size and overall is on a par with the 3D-1p approach. The reproducibility of these results for a given test subject indicate that the observed differences between subject A and B are linked to individual differences in eye physiology

In Fig. 8, for two subjects we present pupil-size data aggregated at the subject level. More specifically, we show normalized relative pupil size as a function of circularity (see “Data processing & Analysis”). For each subject, data from the left eye is displayed, with solid, dotted, and dashed-dotted lines corresponding to recording sessions performed in the bright environment on three consecutive days (see “Multi-day recordings”). Note, in the bin corresponding to the largest circularity (0.9 < *C* < 1.0), all curves coincide due to normalization. The proposed statistics thus reveals by what fraction pupil size is underestimated/overestimated upon a rotation of the eye away from the recording camera; with a circularity of *C* = 0.4 corresponding to a gaze angle of about 50^∘^ with respect to the camera’s optical axis.

While the major axis (red lines) for both subjects tends to progressively underestimate pupil size with decreasing circularity (up to 7.5% at *C* = 0.4), the pupil radius obtained by the 3D-1p approach (blue lines) tends to increasingly overestimate it. Note, this is in line with the sample-level results presented in the last section (see Fig. 6). In case of the 3D-1p approach, however, the extent of overestimation differs between subjects (about 7.5% for subject A at *C* = 0.4, about 3% for subject B at *C* = 0.4). As for the results obtained by the 3D-4p approach (green lines), for subject A correlation with circularity is negative, for subject B it is positive. More importantly, while overestimation of pupil size for subject A is as low as about 2.5% over the range of circularities shown, for subject B an underestimation of about 4% at *C* = 0.4 can be discerned. In other words, while refraction correction renders measured pupil size for subject A largely gaze-angle independent, for subject B it tends to over-correct.

Note, inter-subject differences between curves obtained with the same approach are more pronounced than intra-subject differences for corresponding curves recorded on different days. Our results thus strongly suggest, while day-to-day variations in hardware setup (camera adjustment, pose of the eye tracker on the head of the subject, etc.) modulate results slightly, the observed gaze-angle dependency is predominantly shaped by subject-specific factors such as eye physiology.

### Population level

As shown in the last two sections, on the sample and subject level none of the three measurement methods guarantees gaze-angle independent measurements of pupil size. In practice, however, pupillometry experiments are most often concerned with measuring pupil size as averaged over a large number of test subjects and repetitions of stimulus presentation, i.e. with gauging pupillary responses at the population level. We therefore continue with a presentation of our results at this level of data aggregation.

**Fig. 9:**
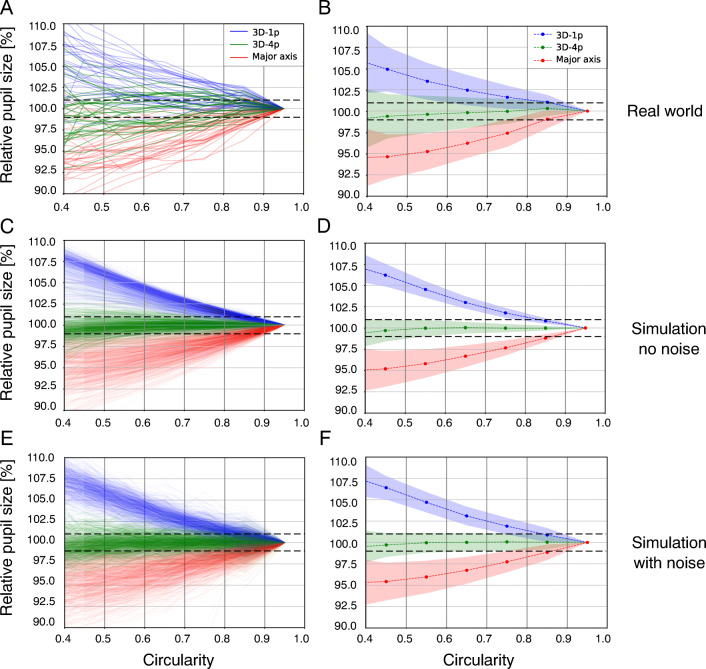
**Population-level results.** Normalized average relative pupil size for all three measurement methods as a function of circularity is shown in (**A**) for all N = 16 subjects (one curve per eye) and for N = 800 simulations runs (one curve per run) in (**C**) *without* pupil-size noise, and in (**E**) *with* pupil-size noise (normally distributed with a standard deviation individually drawn according to the distribution as shown in Fig. 7 (Major axis)). Corresponding mean curves (colored dashed lines) and standard deviations (colored shaded regions) are shown in (**B**), (**D**), and (**F**), respectively. Black dashed lines indicate ± 1*%* deviation from unity

To this end, in Fig. 9A for all subjects, we show subject-level curves of normalized average relative pupil size as a function of circularity. Note, since both the left and right eye was recorded, each subject contributes two curves. In line with results discussed in previous sections, this data shows that the major axis tends to underestimate pupil size with decreasing circularity, while 3D-1p tends to overestimate it. The refraction-corrected 3D-4p approach exhibits over-/underestimation, depending on subject and eye.

Mean curves as calculated per circularity bin are shown for each measurement method in Fig. 9B. Standard deviations around the mean are indicated by shaded regions. While the major axis and 3D-1p show deviations of more than 5 % at C = 0.4 (albeit with opposite sign), the results obtained with 3D-4p stay within ± 1 % (dashed horizontal lines) of the reference value at largest circularity. These results clearly demonstrate that the refraction-aware approach by Dierkes et al., here referred to as 3D-4p, is successful in providing pupil-size measurements which are independent of gaze angle at the population level. In contrast, the major axis and the approach by Świrksi et al., here referred to as 3D-1p, show a similar level of gaze-angle dependency.

In Fig. 9C, D (no pupil-size noise) and Fig. 9E, and F (with pupil-size noise), we present analogous data aggregations based on simulations performed with our raytracing system (see “Experiments - Synthetic data”). We deal with the noiseless case first. In particular, each curve in Fig. 9C reflects a random choice of plausible parameters characterizing eyeball physiology and setup of the eye tracker. These results show that at the population level, the extent of gaze-angle dependency observed in our real-world measurements is in quantitative accord with theoretical predictions for all three measurement methods. Our simulation results also give an indication as to the expected inter-subject variability of gaze-angle dependency. We find that standard deviations measured in our real-world experiments are larger than in simulations (cf. Figure 9B and D), i.e. only part of the observed variability can be explained by reference to variation in physiological eye parameters.

When incorporating pupil-size noise into our simulations, population averages are largely unchanged (cf. Fig. 9D and F). This observation provides evidence that our aggregation scheme is indeed successful in averaging out the effect of unavoidable pupil-size fluctuations (see Fig. 7). At the same time, subject level curves clearly show fluctuations more similar to our real-world data (cf. Fig. 9A and E). Overall, the observed standard deviations, while slightly larger when adding noise in comparison to the noiseless case, are still lower than those of the real-world data (cf. Fig. 9B, D, and F).

We speculate that the remaining variation can be accounted for by measurement noise in the real-world data, resulting inaccuracies in the estimates of eye sphere and eyeball position, and physiological variation in actual eyes that is not captured by the LeGrand eye model.

## Discussion

In this work, we have investigated the extent of PFE-induced gaze-angle dependency in three methods for pupil-size measurement in the context of head-mounted eye tracking. Next to an image-immanent method (major axis), we have considered two model-based approaches (3D-1p, 3D-4p). Presenting both experimental real-world data as well as results from a simulation study, we have analyzed the correlation of pupil-size estimates with gaze angle at the sample, subject, and population level.

Our two experimental setups were carefully designed to minimize pupil-size changes occurring over the time course of each recording, i.e. to approximate constant pupil-size scenarios. This did not, however, prevent physiologically unavoidable pupil-size fluctuations with an amplitude comparable to the effects to be measured. Such pupil-size changes constitute a potentially confounding factor for the assessment of PFE-induced gaze-angle dependency.

A viable strategy for reducing pupil-size fluctuations even further would be the application of a mydriatic drug such as Cyclopentolate that produces sustained pupil dilation. Such an approach, however, constitutes an invasive procedure requiring medical supervision and would furthermore limit experiments to large pupil sizes.

Our analysis instead hinges on the extensive averaging of pupil-size data in terms of 2D pupil circularity, both at the subject as well as the population level. At the resolution of the employed near-eye camera, 50 pixels is a common major axis length of the corresponding 2D pupil in the bright environment (see Fig. 5). A 2 % change in pupil size - a relevant effect in the domain of pupillometry for cognitive sciences - thus corresponds to merely 1 pixel. The proposed averaging strategy is well suited for uncovering such small but systematic dependencies, as it not only combines data from different time points but also from different but equivalent gaze directions. In particular, since pupil-size fluctuations under even illumination are independent of gaze direction, our approach largely eliminates the potentially confounding effect of non-constant pupil sizes. We have further validated the robustness of our approach by incorporating pupil-size fluctuations of realistic amplitude in simulations of synthetic eye images mimicking the performed real-world experiments.

Of all three measurement methods, only the refraction-aware 3D-4p approach resulted in pupil-size estimates that were gaze-angle independent at the population level. Both other approaches were found to exhibit systematic errors at this level of data aggregation. More specifically, with decreasing circularity, pupil sizes tend to be either underestimated (major axis) or overestimated (3D-1p).

Our strategy of aggregating pupil size as a function of circularity suggests an approach for developing a post-hoc correction scheme. By fitting the observed population-level dependency and applying a corresponding multiplicative correction to measured pupil radii in a circularity-dependent manner, the gaze-angle dependency of population averages for the major axis and 3D-1p approach potentially could be reduced.

Even assuming the viability of such post-hoc correction, however, the 3D-4p approach provides advantages. Most importantly, it is not necessitating any prior measurements mapping out systematic errors in pupil-size estimates as a function of circularity. Both model-based approaches are superior to the major axis in that they provide estimates of the actual physical dimensions of the ocular aperture stop in [mm]. At the same time, they also provide estimates of eyeball position and gaze direction, thus integrating pupil-size estimation into a system of broader scope. Since the 3D-4p approach is expected to provide more accurate results in absolute terms than the 3D-1p approach (Dierkes et al., 2019), we overall believe it to be the most convincing choice for many use cases.

Within the cognitive sciences, it is common practice to average pupillometric results at the population level to arrive at statistically meaningful results, due to the small magnitude of effects to be measured, the differences in individual eye physiology as well as reaction to stimuli, and the noisy nature of the underlying video or image data. Our work provides evidence as to the effectiveness of such averaging strategy in the case of head-mounted eye trackers and gives a quantitative estimate of expected systematic errors depending on the measurement approach chosen.

At the sample and subject level, in contrast, all three methods exhibit gaze-angle dependency to some extent. Subject-level curves are spread around corresponding population averages, with the observed spread increasing with decreasing circularity. The observed spread relative to the observed systematic error at population level is similar for all approaches. In particular, while largely being free of systematic errors at the population level, also the 3D-4p approach fails to provide PFE-free pupil-size measurements at the subject level.

Our data shows that gaze-angle dependency on the subject level is largely consistent between recordings obtained on different days, thus strongly suggesting that it is individual variations in eye physiology shaping the observed extent of PFE in each subject. All three approaches are ultimately based on an analysis of the 2D pupil contour. Given a 3D pupil, the exact shape of the 2D pupil image depends on person-specific parameters, e.g. corneal radius, which are determining the optical characteristics of the eye. It thus stands to reason that any approach aiming at PFE-free pupil-size measurement on the subject level, ultimately needs to account for individual differences in eye physiology.

A conceivable post-hoc correction approach would be to map out the dependence of pupil size on circularity for a given subject in a set of experiments such as the ones performed in this study. Using the observed relation as de facto calibration, subject-level results could be corrected by applying a fitted multiplicative factor in a circularity-dependant manner.

Note, the current implementation of the 3D-4p approach assumes average human eye parameters. It is thus plausible that person-specific physiological deviations from those averages - together with particularities in the eye physiology which are not modelled at all - will lead to imperfect correction of pupil-size measurements on the subject level. A potentially less time-intensive and more principled approach would thus be to make person-specific parameters in the 3D-4p approach adjustable, in order to account for the specifics of a subject’s eye physiology. While raytracing synthetic images for a given parameter set and deriving corresponding refraction functions is feasible, it remains an open question, however, whether relevant ocular parameters could be measured easily with sufficient accuracy in order for this approach to be practical.

Also note, the LeGrand eye model constitutes an approximation of ocular optics, as it does not take into account e.g. the effect of non-sphericity of the cornea, variations in corneal thickness, non-circularity of the pupil, or asymmetries in eyeball shape. Facilitating pupil-size measurement which is truly gaze-angle independent on the subject level might thus also necessitate the use of more realistic eye models, such as the Navarro eye model, albeit at the cost of an increased number of person-specific parameters to be determined. Since raytracing is also feasible for eye models of increased veracity, we believe, however, the general approach of determining refraction-correction functions in a person-specific manner by means of synthetic eye images to be a promising strategy.

## Footnotes

1 The image of the pupil is in fact not an exact ellipse, as e.g. pointed out in Fedtke et al., (2010): “[...] the shape of the peripheral entrance pupil does not correspond to an ellipse as often assumed. Instead, although mathematically different, it resembles the shape of a convex https://en.wikipedia.org/wiki/Limacon”. In the following, however, we will disregard this technicality and refer to the pupil image as being an ellipse.

## Declarations

Both authors are employees of Pupil Labs, Berlin, Germany. No funds, grants, or other support was received. All test subjects were employees of Pupil Labs and consented to partake in the study. None of the experiments was preregistered. The data and materials as well as example code which implements all data analysis steps described herein can be accessed under (Pupil Labs, 2021). Open source packages used for data analysis are referenced.
